# Intergenerational Reminiscence Approach in Improving Emotional Well-Being of Older Asian Americans in Early-Stage Dementia Using Virtual Reality: Protocol for an Explanatory Sequential Mixed Methods Study

**DOI:** 10.2196/48927

**Published:** 2023-06-26

**Authors:** Ling Xu, Aaron Hagedorn, Iris Chi

**Affiliations:** 1 School of Social Work University of Texas at Arlington Arlington, TX United States; 2 Suzanne Dworak-Peck School of Social Work University of Southern California Los Angeles, CA United States

**Keywords:** intergenerational reminiscence, grandparent-grandchild, dyad, emotional, well-being, Asian American, older adults, dementia, virtual reality, VR

## Abstract

**Background:**

After a dementia diagnosis, Asian Americans experience anxiety, feelings of shame, and other negative effects. Emotional well-being is not only an important aspect of mental health, but also a quality of resilience that helps people bounce back faster from difficulties. However, few studies have addressed issues in developing, implementing, and testing intervention strategies to promote emotional well-being among older adults. Intergenerational solidarity between grandparents and grandchildren has been emphasized in Asian families and is beneficial for the health of persons with dementia. Reminiscence and life review have been identified as potentially effective intervention strategies for helping depression and emotional well-being for older adults.

**Objective:**

This proposed study aims to develop and implement an intergenerational reminiscence approach and evaluate its potential feasibility and effectiveness in improving the emotional well-being of older Asian American adults who have a recent dementia diagnosis.

**Methods:**

An explanatory sequential mixed methods design will be used in which quantitative data will first be collected and analyzed to identify subsamples of participants who report the greatest and least change in emotional well-being; then, these subsamples will be interviewed to further understand why or why not this intervention works for them. Older adults will receive 6 sessions of life review with grandchildren in virtual reality (VR; 1-1.5 hours each week for 6 weeks), aided by pictures and virtually traveling to important places in their life using Google Earth to look around at those places and remember important times. Quantitative survey data will be collected pre- and postintervention and at a 3-month follow-up. Qualitative interviews with selected participants will also be integrated into the study design. The quantitative data from the surveys will be entered into SPSS (IBM Corp) and analyzed using descriptive analyses, Pearson chi-square tests, nonparametric Friedman tests, or nonparametric Wilcox signed-rank tests (2-tailed). The qualitative data will be transcribed by research assistants, coded by the investigators independently, and analyzed with guidance from content analysis software (Atlas.ti; Atlas.ti Scientific Software Development GmbH).

**Results:**

The project was delayed due to the COVID-19 pandemic. Data collection started in late 2021, and 26 participants were recruited as of December 2022. While we are still cleaning and analyzing the quantitative data, the qualitative interviews showed promising results of this intergenerational reminiscence approach in improving emotional well-being among older Asian American adults who have cognitive impairment.

**Conclusions:**

Intergenerational reminiscence provided by grandchildren is promising in improving the emotional well-being of grandparents. VR technology is likely to be accepted by older adults. Future research may consider scaling up this pilot into a trackable, replicable model that includes more participants and develops a more rigorous study design with control groups to test the effectiveness of this intervention for older adults with dementia.

**International Registered Report Identifier (IRRID):**

DERR1-10.2196/48927

## Introduction

### Background

The Asian American population in the United States nearly doubled between 2000 and 2019. Asians now make up about 7% of the nation’s overall population, and their numbers are projected to surpass 46 million by 2060, nearly four times their current total [1]. Older Asian immigrants often immigrate to the United States at a late age [2] and experience many challenges related to migration, such as low acculturation, limited English language proficiency, ineligibility for entitlement and welfare programs, family or generational conflicts, and social isolation [3,4]. Older Asian American adults also face numerous challenges and physical, psychological, and mental health problems [5], as well as racial discrimination on a regular basis, especially after COVID-19 [6,7]. As the US population becomes older and more diverse, programs and interventions on how to improve mental health of older immigrants are sorely needed.

In addition, if older immigrants are diagnosed with dementia, they face triple adversity: immigration, aging, and dementia. Immigration is a life-changing event that is often accompanied by elevated stress and adversity; aging adds an additional layer of adversity to immigration, and dementia can lead to experiencing further adverse events. This triple jeopardy poses a trauma risk to older Asian American adults, creating a pressing need to understand how to best restore and preserve their resilience. After a dementia diagnosis, people often experience anxiety, negative impacts on self-esteem, preoccupation with the diagnosis, and hyper-vigilance, which can precipitate a crisis [8]. Asian Americans also report feelings of shame and weakness after a dementia diagnosis; however, they seek fewer formal services for support compared to Whites [9-11], leading to negative emotional and mental health outcomes [12]. Despite vulnerabilities among this population, Asian Americans often attract less research attention, in part due to the “model minority” myth [13].

Despite their overall better physical health, older Asian immigrants have worse mental health outcomes than their White counterparts [14-16]. People who are emotionally well (ie, are satisfied with life, have a positive affect, and find purpose or meaning in life) have a quality of resilience and are able to bounce back faster from difficulties [17]. While there are some studies that have specifically looked at emotional well-being among adults and adolescents [18], few studies have examined emotional well-being among older adults. Prevention and interventions to improve emotional well-being among aging populations have attracted special attention because of its strong correlation with health [19]. Reminiscence and life review have been identified as potentially effective intervention strategies for helping depression and increasing emotional well-being among older adults [20-23]. The literature also asserts the positive impact of an intergenerational approach in achieving positive mental health and emotional well-being among older adults [24-26]. Intergenerational solidarity between grandparents and grandchildren has been emphasized in Asian families [27] and is beneficial for the health of persons with dementia [28,29]. When reminiscence and life review strategies are combined with the intergenerational approach, the mental health benefits for older adults are even clearer and more significant [30]. This approach may also help grandchildren gain more historical and cultural understanding of their grandparents’ lived experiences, positively change their views of aging, and increase their bonding with grandparents.

Using technology to help enhance the well-being of older adults is a growing research and practice trend. One recent technological advancement is audiovisual virtual reality (VR), which creates a 3D computer-generated environment to provide an immersive and interactive personal experience [31]. Emerging technologies that allow older adults to socialize across geographic boundaries using a head-mounted display paired with a smartphone have been well accepted by older adults [32]. Studies have also shown the potential of social VR as a powerful reminiscence tool to challenge stereotypes of aging and promote the well-being of older adults [33].

### Aims and Research Questions

This proposed study aims to develop and evaluate the potential feasibility and effectiveness of an intergenerational reminiscence approach in improving the emotional well-being of older Asian American adults who have a recent dementia diagnosis. This pilot study is the first to develop and test how social VR can help older Asian Americans improve their emotional well-being and cope with the stress and adversity of having been diagnosed with dementia. It is hypothesized that the resiliencies of family through grandchildren, reminiscence, and technology together will help older adults develop better emotional well-being, which will help them withstand difficulties in the journey of dementia and have better mental health outcomes. The study addresses three specific aims: (1) develop 6 intervention sessions based on the reminiscence and life review theory using VR technology; (2) quantitatively test the effectiveness of this intervention in improving emotional well-being; and (3) qualitatively evaluate the usefulness of this intervention.

## Methods

### Study Design

An explanatory sequential mixed methods design [34] will be used, involving first collecting quantitative data and then analyzing this data to identify subsamples of participants who report the greatest and least change in emotional well-being. Next, we will conduct qualitative interviews among these subsamples. For the intervention, a single-arm trial will be used in which all the recruited participants are given the intervention and then followed over time to observe their response. This design is used when the objective of the trial is to obtain preliminary evidence of the efficacy of the intervention, but it is not generally used for confirmation of efficacy [35]. Such a design may be desirable when the available participant pool is limited; thus, it is not optimal to randomize many participants to a control arm [35].

### Recruitment

Participants in this study will be recruited from immigrant Chinese, Korean, Vietnamese, and Indian families since they are the top 4 minority Asian groups in the greater urban area of Dallas, Texas [36]. The eligibility criteria of the older adult participants include (1) self-identification as Chinese, Korean, Vietnamese, or Indian American and living in the greater Dallas area; (2) age 65 years or older; (3) a dementia diagnosis in the past 12 months (self-reported); and (4) at least one grandchild aged 18 years or older. When both an eligible older adult and his or her grandchild agree to participate in the study, the grandparent-grandchild dyad will be included in the study. The dyads will be recruited through multiple ways, including referrals from facilities or community agencies, flyers posted in Asian grocery markets, announcements posted on Facebook and Twitter, and local churches that service these Asian American groups. Eight dyads from each of the 4 Asian groups are proposed to be recruited (64 total participants).

### Procedure

After participants sign the informed consent form, both the older adults and their grandchildren will be offered a VR headset. Training on how to use the VR headset will be offered to grandparents and grandchildren by the research team before the intervention starts. If the grandchildren do not reside locally, Microsoft Teams–based training will be offered. Then, older adults will receive 6 sessions of life review with their grandchildren in VR (1-1.5 hours each week for 6 weeks). The themes will include (1) major turning points in life, (2) family history, (3) life and career accomplishments, (4) history of loves and hates, (5) experiences of suffering or stressful experiences, and (6) meaning and purpose of life. A manual of interaction guidelines will be prepared for the grandchildren to lead the interactions with their grandparents based on the intervention manual proposed by Watt and Cappeliez [37,38] and others, which will outline the implementation of integrative and instrumental reminiscence interventions that promote acceptance of self, conflict resolution and reconciliation, a sense of meaning and self-worth, recalling how one coped with past problems, and drawing from past experience to solve present problems.

The process is a series of discussions using the above themes of major moments in the participants’ lives, aided by pictures and virtually traveling to important places in their lives with Google Earth to look around these places and remember important times. Information on important pictures and places will be collected by the grandchildren before the session by asking the grandparents’ spouses, their own parents, or the grandparents’ caregivers or legally authorized representatives (LARs). Both the grandparents and their grandchildren will participate in the intervention remotely. The VR intervention process is linked to YouTube 360 slideshows, an application that enables 2 or more people to communicate verbally while seemingly in the same shared space despite being physically apart. Participants open the YouTube app and select the video we have created. The videos are slideshows using 360-degree scenery that fits with their personal history and the images provided by their family members. The subjects speak over a speakerphone, talking about their memories, while viewing the same video. The intervention through VR linked to YouTube is not recorded, and the private family account set up for this study does not publicly list any scenery video and is thus private to the participants (grandparents and grandchildren), the older participants’ caregivers or LARs, and the research team. Mobile Oculus Quest headsets will be mailed to each participant in the dyads. These headsets do not require a computer and enable an immersive video chat–like experience where participants can do activities together in a virtual world. Some older adults with dementia may need some help from their caregivers or LARs if needed. An eyeglass spacer (a rubber border) comes with every headset provided to the subjects. The interaction process via VR will offer older adults audio as well as visual perceptions and thus increase interactions and engagements while overcoming the limitations of geographical distance between the 2 generations. Pretest, posttest, and 3-month follow-up tests will be performed. Qualitative interviews with selected participants will also be integrated into the study design.

### Measurement

The primary outcomes of the study will be the social and emotional well-being of grandparents, and the secondary outcomes are the benefits to the grandchildren.

#### Primary Outcome Measurements

Primary outcomes among the grandparents include satisfaction with life, emotional well-being, subjective happiness, resilience, depression, and grandparent-grandchild bonds; these are assessed via culturally appropriate standardized instruments before and after the intervention. Demographic information for each dyad, such as age, gender, education, geographic proximity, and self-rated health, are also collected in the pretest survey.

Satisfaction with life is measured by the Satisfaction with Life Scale (SWLS) [39], a 5-item scale designed to measure global cognitive judgments of one’s life satisfaction as a whole. Participants indicate how strongly they agree or disagree with each of the 5 items using a 7-point scale that ranges from 7 (strongly agree) to 1 (strongly disagree). The SWLS has shown good reliability and validity and thus has been recommended as a complement to scales that focus on psychopathology or emotional well-being because it assesses individuals’ conscious evaluative judgment of their life by using their own criteria [40].

Emotional well-being will be measured by the Warwick-Edinburgh Mental Wellbeing Scale (WEMWBS) [41], which consists of 14 items that describe participants’ experience over the past 2 weeks. The WEMWBS covers positive affect (feelings of optimism, cheerfulness, and relaxation), satisfying interpersonal relationships, and positive functioning (energy, clear thinking, self-acceptance, personal development, competence, and autonomy). Each item is measured using a 5-point Likert scale (“none of the time,” “rarely,” “some of the time,” “often,” and “all of the time). Sum scores were calculated with a range of 14 to 70, with a higher score indicating a higher level of mental well-being. The WEMWBS has shown good reliability and validity [41].

The Subjective Happiness Scale (SHS) [42] is a global subjective assessment of whether a person is happy or unhappy. The SHS consists of 4 items, each measured on a 7-point Likert scale ranging from 1 to 7. Sum scores of the 4 items are calculated, yielding a range from 7 to 28. The SHS has been translated to different languages and has shown good reliability and validity.

Resilience of older adults will be measured by the Brief Resilience Scale (BRS) [43,44]. The BRS is one of the best and most highly recommended [44] resilience scales and has shown high reliability and validity in the literature [43,45,46]. Six items are used, each ranging from 1 (low resilience) to 5 (high resilience). After reverse coding 3 items, sum scores are calculated for the final analysis, with a higher score indicating more resilience.

The Geriatric Depression Scale (GDS) [47] short form consists of 15 questions that ask participants to answer yes or no in reference to how they felt over the past week. Of the 15 items, 10 indicate the presence of depression when answered positively while the rest indicate depression when answered negatively. The GDS-15 has been tested and used extensively with the older population and is easy to administer (it takes about 5 to 7 minutes to complete).

Grandparent-grandchild bonds will be measured by 3 questions assessing the quality of their relationship using a scale adapted from the Intergenerational Solidarity Inventory [48]. The 3 questions are as follows: (1) “Taking everything into consideration, how close do you feel to this grandchild?” (2) “How much do you feel that this grandchild would be willing to listen when you need to talk about your worries and problems?” and (3) “Overall, how well do you and this grandchild get along together?” For each question, scores range from 0 to 2 (0=“not at all,” 1=“somewhat,” 2=“very”). An additive emotional cohesion score was computed, ranging from 0 to 6, with higher scores representing a closer grandparent-grandchild relationship.

Usability of virtual reality technology will be measured by adapting the System Usability Scale (SUS) [49]. The SUS provides a “quick and dirty” but reliable tool for measuring usability. It consists of a 10-item questionnaire with 5 response options (from “strongly agree” to “strongly disagree”). The SUS allows researchers to evaluate a wide variety of products and services, including hardware, software, mobile devices, websites, and applications [50]. In this study, we added “VR” to the 10 statements. In addition, 3 separate questions are asked to evaluate participants’ subjective feelings on VR technology (whether they find it comfortable, satisfactory, and effective), with “yes”, “no,” and “unsure” answers for each question.

#### Secondary Outcome Measurements

Secondary outcome measurements for the grandchildren include emotional well-being, attitude toward aging, the grandparent-grandchild relationship, and the usability of VR technology. Except for the attitude toward aging, all measurements for the grandchildren will be the same as those for the grandparents.

The young adult volunteers’ attitude toward aging will be measured with the Fraboni Scale of Ageism (FSA), which was developed by Fraboni et al [51] and revised by Rupp et al [52]. Participants will be asked to respond on a 4-point Likert scale to 29 statements (1=“strongly disagree” to 4=“strongly agree”). The FSA has been extensively used among college students and has shown high internal consistency and strong validity [53,54]. Sum scores will be calculated for the final analysis, with higher scores indicating more ageism.

#### Other Outcomes

Follow-up qualitative interviews are also conducted to explore participants’ experience with VR technology and their overall experience and feelings regarding the proposed intervention. We will select 3 dyads from each group for further qualitative interviews (12 dyads total). Each generation’s experiences, perspectives, challenges, and suggestions with this intervention will be determined with in-depth interviews (in person or via a phone call lasting for about 45 to 60 minutes). In general, we will use the following probe questions for the in-depth interviews with older adults: (1) “In general, what did you share with your grandchild in the past few weeks?” (2) “Can you describe how your emotional well-being has changed as a result of the intervention program? Please provide a few examples, especially on what part of the intervention helped you most or least when improving your well-being” (3) “How did you feel about the weekly talk with your grandchild?” (4) “How do you feel about the virtual reality technology in this intervention program?” and (5) “What suggestions or advice do you have for this project? Or any suggestion or advice for improving your emotional well-being?” For the grandchildren, we will use the following questions: (1) “In general, what did you talk with your grandparent in the past few weeks? Or what contents did you share with each other?” (2) “Can you describe how your grandparent’s emotional well-being has changed as a result of the intervention program? Please provide a few examples, especially what part of the intervention helped you most when improving your well-being” (3) “How did you feel about the weekly talk with your grandparent? Challenges? Ways to overcome?” (4) “How do you feel about the virtual reality technology in this intervention program?” and (5) “What suggestions or advice do you have for this project? Or any suggestion or advice for improving the emotional well-being of your grandparent?”

### Statistical Analysis

The quantitative data from surveys will be entered into SPSS (IBM Corp) and analyzed using descriptive analyses, Pearson chi-square tests, Friedman tests, or nonparametric Wilcox signed-rank tests (2-tailed). The qualitative data will be transcribed by research assistants, coded by the investigators independently, and then analyzed with the guidance of content analysis software (Atlas.ti; Atlas.ti Scientific Software Development GmbH).

### Ethical Considerations

Prior to commencing the study, all members of the research team successfully completed the Human Subject Protection training and acquired the necessary certification. The study adheres strictly to the guidelines outlined in the Declaration of Helsinki and received approval on July 14, 2020, from the University of Texas at Arlington (UTA) institutional review board (2020-0210). Before conducting the interviews, the participants were provided with clear and comprehensive information regarding the purpose of the study, the procedures involved, the potential risks and benefits associated with participation, the use and anonymization of research data, and the maintenance of confidentiality. Furthermore, the participants were explicitly informed, both initially and through reminders, that their involvement was completely voluntary and that they could withdraw from the project at any point without facing any penalties. The study officially commenced only after participants signed the consent form. The consent form included the contact details of the principal investigator and the institutional review board office, and each participant received a signed copy for their records. Each participant who successfully completes the study is provided compensation in the form of a $110 Walmart gift card.

Each participant is given an ID number. The data obtained for this study has been carefully collected to exclude any personal identifiers. All data files are securely stored in password-protected and encrypted formats on laptops provided by the UTA. These UTA-issued laptops also adhere to encryption protocols mandated by the UTA Office of Information Technology. The encrypted data files will be stored using an approved UTA storage tool, such as OneDrive, to ensure their secure preservation. The utmost priority is given to safeguarding the privacy of the participants involved. A list containing participant names will be maintained securely using author LX’s authorized and protected OneDrive account. In the final report, only deidentified data will be included, ensuring anonymity. The data will be retained for a period of 3 years following the conclusion of the study’s protocol.

## Results

This study was funded by the Rutgers University Asian Resource Centers for Minority Aging Research Center under the National Institutes of Health/National Institute on Aging (grant P30-AG0059304) for a project duration of April 4, 2020, to June 30, 2023. The project was delayed due to the COVID-19 pandemic. Data collection started in late 2021. As of December 2022, 26 participant dyads were recruited, for a response rate of approximately 72% (we approached 36 potential dyads). Among the 26 grandchild participants, 11 were Chinese American, 8 were Vietnamese American, 5 were Indian American and 2 were Korean American. Data for the grandparents at baseline and after the intervention were collected by phone calls by trained bilingual research assistants. Individual grandchild participants entered the data in QuestionPro (Survey Analytics LLC), a centrally supported online survey research tool. The data collected were checked for correctness by means of cross-validation.

After the intergenerational reminiscence intervention was completed, 11 dyads were selected for further qualitative interviews. However, the majority of the dyads interviewed were from Chinese American families. We are currently in the stage of entering, cleaning, and analyzing the quantitative and qualitative data.

## Discussion

### Principal Findings

The objective of this study is to create and implement a 6-session intervention using VR technology and the reminiscence approach targeting grandparents and grandchildren in Asian American families. By gathering both qualitative and quantitative data, this study aims to assess the effectiveness of the intervention in enhancing the emotional well-being of grandparents and strengthening intergenerational bonds. The research will contribute valuable and up-to-date data to address existing research gaps, particularly by focusing on reminiscence and life review content, incorporating VR technology, and including Asian grandparents who experience cognitive impairment.

Given the various physical, psychological, and mental health issues and instances of discrimination faced by Asian American families [5-7], this study holds significant importance. By focusing on the emotional well-being of older Asian adults, it aims to enhance their life satisfaction, positive emotions, and sense of purpose, all of which contribute to resilience and faster recovery from adversities [17], including the challenges associated with dementia diagnosis. Consequently, this study makes a valuable contribution to the limited existing literature on emotional well-being among older adults, particularly within the context of Asian American families.

Moreover, this research incorporates an innovative approach that combines the positive practice of reminiscence and life review [20-23] with an intergenerational perspective [24-26]. While intergenerational approaches involving younger and older generations have been documented in the literature [24-26], it is worth noting that no previous studies, to the best of the authors’ knowledge, have applied this approach specifically to grandparents and grandchildren. This grandparent-grandchild approach holds special significance for Asian Americans, as it aligns with the emphasis placed on intergenerational solidarity between grandparents and grandchildren within their culture. By engaging in this approach, grandchildren can gain a deeper understanding of their immigrant grandparents’ lived experiences, from both a historical and cultural perspective. This, in turn, has the potential to positively reshape their perceptions of aging and strengthen their bonds with their grandparents.

### Strengths and Limitations

This mixed methods study is the first to develop and examine an intergenerational reminiscence-based intervention to improve the well-being of older adults in Asian American families with the aid of VR technology. Intergenerational reminiscence provided by grandchildren is a promising approach to improving the emotional well-being of grandparents, as VR technology is likely to be accepted by older adults. This study will help increase intergenerational closeness between grandparents and grandchildren. Findings from this study will also help health or human service providers develop programs to help Asian American families in general.

Limitations of this study include its small sample size and the absence of a control group. Future research may consider scaling up this pilot into a trackable, replicable model that includes more participants and has a more rigorous study design with a control group to test the effectiveness of this intervention for older adults with dementia. In addition, because there are wide differences between different Asian-American groups, it would be better to focus on only one Asian ethnic minority for one research project and compare the differences within that ethnic Asian group. Lastly, this protocol is proposed for in-person interactions between grandparents and grandchildren. Due to the risks and feasibility of conducting an in-person intervention during COVID-19, this project had to be tailored to a telephone-based approach. Future studies may consider comparing the effectiveness of interventions delivered as in-person meetings versus those that use a telephone-based approach.

### Conclusions

Intergenerational reminiscence programs have the potential to foster social connection and engagement between grandparents and grandchildren within Asian American families. These programs also serve as a means for grandchildren to gain a deeper understanding of the life experiences of older adults both before and after immigration. By specifically focusing on intergenerational reminiscence between grandparents and grandchildren, a valuable avenue is opened for strengthening generational bonding and relationships. This, in turn, has the potential to enhance the psychological well-being, particularly the emotional well-being, of older Asian immigrant adults residing in the United States. Furthermore, this study will contribute valuable evidence to support future research exploring the feasibility and effectiveness of using VR in interventions or programs designed for older adults with cognitive impairments.

## Appendix

Multimedia Appendix 1 Peer-review reports from the Rutgers Institute for Health, Health Care Policy and Aging Research, The State University of New Jersey (United States).

## Abbreviations

BRS: Brief Resilience Scale
FSA: Fraboni Scale of Ageism
GDS: Geriatric Depression Scale
LAR: legally authorized representative
SHS: Subjective Happiness Scale
SUS: System Usability Scale
SWLS: Satisfaction with Life Scale
UTA: University of Texas at Arlington
VR: virtual reality
WEMWBS: Warwick-Edinburgh Mental Wellbeing Scales

## Data Availability

The data sets generated during and/or analyzed during this study are available from the corresponding author on reasonable request.
