# Supplementary material for: Intergenerational Reminiscence Approach in Improving Emotional Well-Being of Older Asian Americans in Early-Stage Dementia Using Virtual Reality: Protocol for an Explanatory Sequential Mixed Methods Study
Source: JMIR Res Protoc. 2023 Jun 26;12:e48927. doi: 10.2196/48927 (PMC10337457; doi:10.2196/48927)
Supplement: Multimedia Appendix 1 [file resprot_v12i1e48927_app1.pdf]

RUTGERS
Institute for Health, Health Care Policy, and Aging Research

Institute for Health, Health Care Policy and Aging Research
Rutgers, The State University of New Jersey
112 Paterson Street
New Brunswick, NJ 08901

ifh.rutgers.edu
Phone: 848-932-8413
Fax: 732-932-1253

Evaluation Form for RCMAR Pilot Applications

Name of applicant: Ling Xu

Instructions to Reviewers:

For each review criterion below, select the word that best represents your rating of the application for that element by selecting the box provided to the right of that word. After rating the individual criteria, in four or five sentences, summarize your overall impressions of the applicant's potential to remain in a research career.

Finally assign an overall score using the scale from 1 (exceptional) to 9 (Poor).

Review Criteria:

The primary consideration is to assess the potential of the applicant to pursue a career in research.

Overall Score: Choose the number that best indicates your overall rating of the application.

1. Exceptional 2. Outstanding 3. **Excellent** 4. Very Good 5. Good 6. Satisfactory 7. Fair 8. Marginal 9. Poor

A. Potential of the applicant to pursue a career in research

1. Appropriateness of the applicant's previous training and experience to prepare for a research career

   1. Exceptional 2. Outstanding 3. **Excellent** 4. Very Good 5. Good 6. Satisfactory 7. Fair 8. Marginal 9. Poor

2. Appropriateness of the proposed research activities during the one-year RCMAR pilot period to foster a career in research. (Note: do not evaluate the research on its scientific merits per se, but as a vehicle to help the applicant pursue a research career.)

   1. Exceptional 2. Outstanding **3. Excellent** 4. Very Good 5. Good 6. Satisfactory 7. Fair 8. Marginal 9. Poor

3. Commitment to a research career as reflected by the statement of long-term career goals and the plan to achieve those goals

   1. Exceptional 2. Outstanding **3. Excellent** 4. Very Good 5. Good 6. Satisfactory 7. Fair 8. Marginal 9. Poor

B. Scientific Promise and Merits of the Application

1. Significance of the research project and its connection to trauma, resilience, and health outcomes among Asian older adults

   1. Exceptional 2. Outstanding **3. Excellent** 4. Very Good 5. Good 6. Satisfactory 7. Fair 8. Marginal 9. Poor

2. Innovation and potential of the research project to lead to larger research projects

   1. Exceptional 2. Outstanding **3. Excellent** 4. Very Good 5. Good 6. Satisfactory 7. Fair 8. Marginal 9. Poor

Institute for Health, Health Care Policy and Aging Research
Rutgers, The State University of New Jersey
112 Paterson Street
New Brunswick, NJ 08901
ifh.rutgers.edu
Phone: 848-932-8413
Fax: 732-932-1253

3. Scientific merits/approach of the research project

1. Exceptional 2. Outstanding 3. Excellent **4. Very Good** 5. Good 6. Satisfactory 7. Fair 8. Marginal 9. Poor

4. Quality and availability of appropriate scientific mentors and colleagues to help achieve or enhance the applicant's research independence

1. Exceptional 2. Outstanding 3. Excellent **4. Very Good** 5. Good 6. Satisfactory 7. Fair 8. Marginal 9. Poor

C. Potential significance and impact of research on scientific knowledge and the Asian community

1. Exceptional 2. Outstanding **3. Excellent** 4. Very Good 5. Good 6. Satisfactory 7. Fair 8. Marginal 9. Poor

D. **Overall Merit Summary**: (In 4-5 sentences, summarize your assessment of the applicant's potential and likelihood to be successful in a research career. (Click the grey box and start typing)

I like this pilot study proposal in its use of virtual reality headsets as an intervention strategy. To me, this is a unique use of VR as a means to improve a client's emotional well being. I look forward to seeing the results of this project should it be funded. Dr. Xu has a good start on her research career.

RUTGERS
Institute for Health, Health Care Policy, and Aging Research

Institute for Health, Health Care Policy and Aging Research
Rutgers, The State University of New Jersey
112 Paterson Street
New Brunswick, NJ 08901

ifh.rutgers.edu
Phone: 848-932-8413
Fax: 732-932-1253

Evaluation Form for RCMAR Pilot Applications

Name of applicant: **Ling Xu**

Instructions to Reviewers:

For each review criterion below, select the word that best represents your rating of the application for that element by selecting the box provided to the right of that word. After rating the individual criteria, in four or five sentences, summarize your overall impressions of the applicant's potential to remain in a research career.

Finally assign an overall score using the scale from 1 (exceptional) to 9 (Poor).

Review Criteria:

The primary consideration is to assess the potential of the applicant to pursue a career in research.

Overall Score: Choose the number that best indicates your overall rating of the application.

1. Exceptional ☐ 2. Outstanding ☐ 3. Excellent ☒ 4. Very Good ☐ 5. Good ☐ 6. Satisfactory ☐ 7. Fair ☐ 8. Marginal ☐ 9. Poor ☐

A. Potential of the applicant to pursue a career in research

1. Appropriateness of the applicant's previous training and experience to prepare for a research career

1. Exceptional ☐ 2. Outstanding ☒ 3. Excellent ☐ 4. Very Good ☐ 5. Good ☐ 6. Satisfactory ☐ 7. Fair ☐ 8. Marginal ☐ 9. Poor ☐

2. Appropriateness of the proposed research activities during the one-year RCMAR pilot period to foster a career in research. (Note: do not evaluate the research on its scientific merits per se, but as a vehicle to help the applicant pursue a research career.)

1. Exceptional ☐ 2. Outstanding ☐ 3. Excellent ☒ 4. Very Good ☐ 5. Good ☐ 6. Satisfactory ☐ 7. Fair ☐ 8. Marginal ☐ 9. Poor ☐

3. Commitment to a research career as reflected by the statement of long-term career goals and the plan to achieve those goals

1. Exceptional ☐ 2. Outstanding ☒ 3. Excellent ☐ 4. Very Good ☐ 5. Good ☐ 6. Satisfactory ☐ 7. Fair ☐ 8. Marginal ☐ 9. Poor ☐

B. Scientific Promise and Merits of the Application

1. Significance of the research project and its connection to trauma, resilience, and health outcomes among Asian older adults

1. Exceptional ☐ 2. Outstanding ☒ 3. Excellent ☐ 4. Very Good ☐ 5. Good ☐ 6. Satisfactory ☐ 7. Fair ☐ 8. Marginal ☐ 9. Poor ☐

2. Innovation and potential of the research project to lead to larger research projects

1. Exceptional ☐ 2. Outstanding ☒ 3. Excellent ☐ 4. Very Good ☐ 5. Good ☐ 6. Satisfactory ☐ 7. Fair ☐ 8. Marginal ☐ 9. Poor ☐

3. Scientific merits/approach of the research project

1. Exceptional ☐ 2. Outstanding ☐ 3. Excellent ☐ 4. Very Good ☒ 5. Good ☐ 6. Satisfactory ☐ 7. Fair ☐ 8. Marginal ☐ 9. Poor ☐

RUTGERS
Institute for Health, Health Care Policy, and Aging Research

Institute for Health, Health Care Policy and Aging Research
Rutgers, The State University of New Jersey
112 Paterson Street
New Brunswick, NJ 08901

ifh.rutgers.edu
Phone: 848-932-8413
Fax: 732-932-1253

4. Quality and availability of appropriate scientific mentors and colleagues to help achieve or enhance the applicant's research independence

1. Exceptional ☐ 2. Outstanding ☐ 3. Excellent ☐ 4. Very Good ☒ 5. Good ☐ 6. Satisfactory ☐ 7. Fair ☐ 8. Marginal ☐ 9. Poor ☐

C. Potential significance and impact of research on scientific knowledge and the Asian community

1. Exceptional ☐ 2. Outstanding ☐ 3. Excellent ☒ 4. Very Good ☐ 5. Good ☐ 6. Satisfactory ☐ 7. Fair ☐ 8. Marginal ☐ 9. Poor ☐

D. **Overall Merit Summary**: (In 4-5 sentences, summarize your assessment of the applicant's potential and likelihood to be successful in a research career. (Click the grey box and start typing)

The applicant proposes a highly innovative approach to increase resiliency and well being among older Asian Americans recently diagnosed with dementia. If successful, the approach could have broad positive, inter-generational effects. There are a few concerns with the feasibility of the proposed research, however, that slightly dampened enthusiasm for the proposal. The proposal to recruit a total of 20 matched older-younger Asian American pairs seems highly ambitious; there should have been plans articulated for alternative approaches if this target is not met (such as partnering with healthcare providers to conduct clinic-based recruitment). There should be some evidence and rationale that a VR intervention would be acceptable to older Asian Americans. The intervention seems inherently biased towards finding a positive effect in that grandparent-grandchild pairs who are already connected and have a strong relationship are more likely to participate. Again, a clinic-based recruitment approach, rather than a volunteer approach, may mitigate this bias. Overall, however, the research is aligned with the applicant's training and research portfolio to date, and pilot funding will serve to help the applicant be successful in a research career.

RUTGERS
Institute for Health, Health Care Policy, and Aging Research

Institute for Health, Health Care Policy and Aging Research
Rutgers, The State University of New Jersey
112 Paterson Street
New Brunswick, NJ 08901

ifh.rutgers.edu
Phone: 848-932-8413
Fax: 732-932-1253

Evaluation Form for RCMAR Pilot Applications

Name of applicant: **Ling Xu**

Instructions to Reviewers:

For each review criterion below, select the word that best represents your rating of the application for that element by selecting the box provided to the right of that word. After rating the individual criteria, in four or five sentences, summarize your overall impressions of the applicant’s potential to remain in a research career.

Finally assign an overall score using the scale from 1 (exceptional) to 9 (Poor).

Review Criteria:

The primary consideration is to assess the potential of the applicant to pursue a career in research.

Overall Score: Choose the number that best indicates your overall rating of the application.

1. Exceptional ☐ 2. Outstanding ☒ 3. Excellent ☐ 4. Very Good ☐ 5. Good ☐ 6. Satisfactory ☐ 7. Fair ☐ 8. Marginal ☐ 9. Poor ☐

A. Potential of the applicant to pursue a career in research

1. Appropriateness of the applicant’s previous training and experience to prepare for a research career

1. Exceptional ☐ 2. Outstanding ☐ 3. Excellent ☒ 4. Very Good ☐ 5. Good ☐ 6. Satisfactory ☐ 7. Fair ☐ 8. Marginal ☐ 9. Poor ☐

2. Appropriateness of the proposed research activities during the one-year RCMAR pilot period to foster a career in research. (Note: do not evaluate the research on its scientific merits per se, but as a vehicle to help the applicant pursue a research career.)

1. Exceptional ☐ 2. Outstanding ☒ 3. Excellent ☐ 4. Very Good ☐ 5. Good ☐ 6. Satisfactory ☐ 7. Fair ☐ 8. Marginal ☐ 9. Poor ☐

3. Commitment to a research career as reflected by the statement of long-term career goals and the plan to achieve those goals

1. Exceptional ☐ 2. Outstanding ☒ 3. Excellent ☐ 4. Very Good ☐ 5. Good ☐ 6. Satisfactory ☐ 7. Fair ☐ 8. Marginal ☐ 9. Poor ☐

B. Scientific Promise and Merits of the Application

1. Significance of the research project and its connection to trauma, resilience, and health outcomes among Asian older adults

1. Exceptional ☐ 2. Outstanding ☐ 3. Excellent ☒ 4. Very Good ☐ 5. Good ☐ 6. Satisfactory ☐ 7. Fair ☐ 8. Marginal ☐ 9. Poor ☐

2. Innovation and potential of the research project to lead to larger research projects

1. Exceptional ☐ 2. Outstanding ☒ 3. Excellent ☐ 4. Very Good ☐ 5. Good ☐ 6. Satisfactory ☐ 7. Fair ☐ 8. Marginal ☐ 9. Poor ☐

3. Scientific merits/approach of the research project

1. Exceptional ☐ 2. Outstanding ☐ 3. Excellent ☒ 4. Very Good ☐ 5. Good ☐ 6. Satisfactory ☐ 7. Fair ☐ 8. Marginal ☐ 9. Poor ☐

Institute for Health, Health Care Policy and Aging Research
Rutgers, The State University of New Jersey
112 Paterson Street
New Brunswick, NJ 08901
ifh.rutgers.edu
Phone: 848-932-8413
Fax: 732-932-1253

4. Quality and availability of appropriate scientific mentors and colleagues to help achieve or enhance the applicant's research independence

1. Exceptional ☐ 2. Outstanding ☐ 3. Excellent ☐ 4. Very Good ☐ 5. Good ☐ 6. Satisfactory ☐ 7. Fair ☐ 8. Marginal ☐ 9. Poor ☐

C. Potential significance and impact of research on scientific knowledge and the Asian community

1. Exceptional ☐ 2. Outstanding ☒ 3. Excellent ☐ 4. Very Good ☐ 5. Good ☐ 6. Satisfactory ☐ 7. Fair ☐ 8. Marginal ☐ 9. Poor ☐

D. **<u>Overall Merit Summary</u>**: (In 4-5 sentences, summarize your assessment of the applicant's potential and likelihood to be successful in a research career. (Click the grey box and start typing)

To develop intergenerational intervention may provide active improvement of health outcome, rather than just collect data.

RUTGERS
Institute for Health, Health Care
Policy, and Aging Research

Institute for Health, Health Care Policy and
Aging Research
Rutgers, The State University of New Jersey
112 Paterson Street
New Brunswick, NJ 08901

ifh.rutgers.edu
Phone: 848-932-8413
Fax: 732-932-1253

Evaluation Form for RCMAR Pilot Applications

Name of applicant: Ling Xu

Instructions to Reviewers:

For each review criterion below, select the word that best represents your rating of the application for that element by selecting the box provided to the right of that word. After rating the individual criteria, in four or five sentences, summarize your overall impressions of the applicant's potential to remain in a research career.

Finally assign an overall score using the scale from 1 (exceptional) to 9 (Poor).

Review Criteria:

The primary consideration is to assess the potential of the applicant to pursue a career in research.

Overall Score: Choose the number that best indicates your overall rating of the application.

1. Exceptional ☒ 2. Outstanding ☐ 3. Excellent ☐ 4. Very Good ☐ 5. Good ☐ 6. Satisfactory ☐ 7. Fair ☐ 8. Marginal ☐ 9. Poor ☐

A. Potential of the applicant to pursue a career in research

1. Appropriateness of the applicant's previous training and experience to prepare for a research career

1. Exceptional ☒ 2. Outstanding ☐ 3. Excellent ☐ 4. Very Good ☐ 5. Good ☐ 6. Satisfactory ☐ 7. Fair ☐ 8. Marginal ☐ 9. Poor ☐

2. Appropriateness of the proposed research activities during the one-year RCMAR pilot period to foster a career in research. (Note: do not evaluate the research on its scientific merits per se, but as a vehicle to help the applicant pursue a research career.)

1. Exceptional ☐ 2. Outstanding ☒ 3. Excellent ☐ 4. Very Good ☐ 5. Good ☐ 6. Satisfactory ☐ 7. Fair ☐ 8. Marginal ☐ 9. Poor ☐

3. Commitment to a research career as reflected by the statement of long-term career goals and the plan to achieve those goals

1. Exceptional ☒ 2. Outstanding ☐ 3. Excellent ☐ 4. Very Good ☐ 5. Good ☐ 6. Satisfactory ☐ 7. Fair ☐ 8. Marginal ☐ 9. Poor ☐

B. Scientific Promise and Merits of the Application

1. Significance of the research project and its connection to trauma, resilience, and health outcomes among Asian older adults

1. Exceptional ☒ 2. Outstanding ☐ 3. Excellent ☐ 4. Very Good ☐ 5. Good ☐ 6. Satisfactory ☐ 7. Fair ☐ 8. Marginal ☐ 9. Poor ☐

2. Innovation and potential of the research project to lead to larger research projects

1. Exceptional ☒ 2. Outstanding ☐ 3. Excellent ☐ 4. Very Good ☐ 5. Good ☐ 6. Satisfactory ☐ 7. Fair ☐ 8. Marginal ☐ 9. Poor ☐

3. Scientific merits/approach of the research project

1. Exceptional ☒ 2. Outstanding ☐ 3. Excellent ☐ 4. Very Good ☐ 5. Good ☐ 6. Satisfactory ☐ 7. Fair ☐ 8. Marginal ☐ 9. Poor ☐

Institute for Health, Health Care Policy and Aging Research
Rutgers, The State University of New Jersey
112 Paterson Street
New Brunswick, NJ 08901

ifh.rutgers.edu
Phone: 848-932-8413
Fax: 732-932-1253

4. Quality and availability of appropriate scientific mentors and colleagues to help achieve or enhance the applicant's research independence

1. Exceptional ☒ 2. Outstanding ☐ 3. Excellent ☐ 4. Very Good ☐ 5. Good ☐ 6. Satisfactory ☐ 7. Fair ☐ 8. Marginal ☐ 9. Poor ☐

C. Potential significance and impact of research on scientific knowledge and the Asian community

1. Exceptional ☒ 2. Outstanding ☐ 3. Excellent ☐ 4. Very Good ☐ 5. Good ☐ 6. Satisfactory ☐ 7. Fair ☐ 8. Marginal ☐ 9. Poor ☐

D. **Overall Merit Summary**: (In 4-5 sentences, summarize your assessment of the applicant's potential and likelihood to be successful in a research career. (Click the grey box and start typing)

The applicant has a solid record of conducting research among older Asian persons, inclusive of an R15 grant from NIH as PI. Her focus has been on intervention studies, including a study involving robotics to assist older persons. The current application expands her portfolio to include the use of virtual reality to connect older Chinese & Korean immigrants with dementia to their grandchildren. The application is exceptionally innovative, responsive to the topics of trauma and resilience, and approaches the project using a theoretical framework. The mentoring team appears solid and bulids on prior relationships & complementary areas of expertise.

RUTGERS
Institute for Health, Health Care Policy, and Aging Research

Institute for Health, Health Care Policy and Aging Research
Rutgers, The State University of New Jersey
112 Paterson Street
New Brunswick, NJ 08901

ifh.rutgers.edu
Phone: 848-932-8413
Fax: 732-932-1253

Evaluation Form for RCMAR Pilot Applications

Name of applicant: Ling Xu

Instructions to Reviewers:

For each review criterion below, select the word that best represents your rating of the application for that element by selecting the box provided to the right of that word. After rating the individual criteria, in four or five sentences, summarize your overall impressions of the applicant's potential to remain in a research career.

Finally assign an overall score using the scale from 1 (exceptional) to 9 (Poor).

Review Criteria:

The primary consideration is to assess the potential of the applicant to pursue a career in research.

Overall Score: Choose the number that best indicates your overall rating of the application.

1. Exceptional ☐ 2. Outstanding ☐ 3. Excellent ☒ 4. Very Good ☐ 5. Good ☐ 6. Satisfactory ☐ 7. Fair ☐ 8. Marginal ☐ 9. Poor ☐

A. Potential of the applicant to pursue a career in research

1. Appropriateness of the applicant's previous training and experience to prepare for a research career

1. Exceptional ☐ 2. Outstanding ☐ 3. Excellent ☐ 4. Very Good ☐ 5. Good ☐ 6. Satisfactory ☐ 7. Fair ☐ 8. Marginal ☐ 9. Poor ☐

2. Appropriateness of the proposed research activities during the one-year RCMAR pilot period to foster a career in research. (Note: do not evaluate the research on its scientific merits per se, but as a vehicle to help the applicant pursue a research career.)

1. Exceptional ☐ 2. Outstanding ☐ 3. Excellent ☐ 4. Very Good ☐ 5. Good ☐ 6. Satisfactory ☐ 7. Fair ☐ 8. Marginal ☐ 9. Poor ☐

3. Commitment to a research career as reflected by the statement of long-term career goals and the plan to achieve those goals

1. Exceptional ☐ 2. Outstanding ☐ 3. Excellent ☐ 4. Very Good ☐ 5. Good ☐ 6. Satisfactory ☐ 7. Fair ☐ 8. Marginal ☐ 9. Poor ☐

B. Scientific Promise and Merits of the Application

1. Significance of the research project and its connection to trauma, resilience, and health outcomes among Asian older adults

1. Exceptional ☐ 2. Outstanding ☐ 3. Excellent ☐ 4. Very Good ☐ 5. Good ☐ 6. Satisfactory ☐ 7. Fair ☐ 8. Marginal ☐ 9. Poor ☐

2. Innovation and potential of the research project to lead to larger research projects

1. Exceptional ☐ 2. Outstanding ☒ 3. Excellent ☐ 4. Very Good ☐ 5. Good ☐ 6. Satisfactory ☐ 7. Fair ☐ 8. Marginal ☐ 9. Poor ☐

3. Scientific merits/approach of the research project
1. Exceptional ☐ 2. Outstanding ☐ 3. Excellent ☐ 4. Very Good ☒ 5. Good ☐ 6. Satisfactory ☐ 7. Fair ☐ 8. Marginal ☐ 9. Poor ☐

4. Quality and availability of appropriate scientific mentors and colleagues to help achieve or enhance the applicant's research independence

1. Exceptional ☐ 2. Outstanding ☒ 3. Excellent ☐ 4. Very Good ☐ 5. Good ☐ 6. Satisfactory ☐ 7. Fair ☐ 8. Marginal ☐ 9. Poor ☐

C. Potential significance and impact of research on scientific knowledge and the Asian community

1. Exceptional ☐ 2. Outstanding ☐ 3. Excellent ☒ 4. Very Good ☐ 5. Good ☐ 6. Satisfactory ☐ 7. Fair ☐ 8. Marginal ☐ 9. Poor ☐

D. **<u>Overall Merit Summary</u>**: (In 4-5 sentences, summarize your assessment of the applicant's potential and likelihood to be successful in a research career. (Click the grey box and start typing)

Strong applicant who is motivated and has an R15 from NIA and whose work is innovative with a focus on using virtual reality technology to promote resilience and intergenerational connection among individuals with dementia and their grandchildren using reminiscience and life review strategies. The intervention is innovative. The study design and feasibility is a concern. It is unclear the exten to which the PI will be able to recruit grandparents and grandchildren dyads. There are two levels of IRB review and questions regarding engagement and recruitment.
